# Bipolar plasma vaporization in secondary bladder neck sclerosis – initial experience with a new technique

**Published:** 2012-03-05

**Authors:** B Geavlete, F Stănescu, Gh Niţă, M Jecu, C Moldoveanu, P Geavlete

**Affiliations:** ”Sf. Ioan” Clinical Emergency Hospital, Department of Urology

**Keywords:** secondary bladder neck sclerosis, bipolar plasma vaporization

## Abstract

**Introduction: **Secondary bladder neck sclerosis (BNS) represents one of the most common long-term complications after prostate surgical treatment. In this retrospective study, we aimed to evaluate our initial experience concerning the bipolar plasma vaporization (BPV) performed in patients with secondary BNS and to assess the efficiency, safety and short-term postoperative results of this approach.

**Materials & Methods: **Between May 2009 and May 2010, a total of 30 male patients with BNS underwent BPV and were followed for a period of 6 months. BNS was secondary to monopolar transurethral resection of prostate (TURP) in 19 cases, to open surgery for BPH (open prostatectomy) in 8 cases and to radical prostatectomy for prostate cancer in 3 cases. The follow-up protocol included the International Prostate Symptom Score (IPSS), quality of life score (QoL), maximum flow rate (Qmax) and post-voiding residual urinary volume (RV) evaluated at 1, 3 and 6 months after surgery.

**Results: **BPV was successfully performed in all cases. All patients were able to void spontaneously and were continent after catheter removal. The mean operating time was 9 minutes, the mean catheterization period was 18 hours and the mean hospital stay was 24 hours. Preoperatively and at 1, 3 and 6 months after surgery, the mean values for Qmax and RV were 7.2 ml/s and 110 ml, 23.9 ml/s and 20 ml, 23.8 ml/s and 28 ml, and 23.4 ml/s and 26 ml, respectively. Before surgery and at 1, 3 and 6 months, the IPSS and QoL scores were 22.6 and 4.1, 3.4 and 1.2, 3.6 and 1.4, and 3.7 and 1.4, respectively.

**Conclusions: **BPV represents a valuable endoscopic treatment alternative for secondary BNS with good efficacy, reduced morbidity, fast postoperative recovery and satisfactory follow-up parameters.

**Abbreviations** BNS – bladder neck sclerosis, BPV – bipolar plasma vaporization, TURP – transurethral resection of the prostate, IPSS – International Prostate Symptom Score, QoL – quality of life score, Qmax – maximum flow rate, RV – post-voiding residual urinary volume

## Introduction

Secondary bladder neck sclerosis (BNS) represents one of the most common long-term complications after the surgical treatment of the prostate. According to the EAU Guidelines, the risk of developing this pathology is 4% after TURP, 1.8% after open surgery for BPH and 0.5-14.6% after radical prostatectomy for prostate cancer [**1**].

BNS management includes periodic dilation, bladder neck incision, standard monopolar transurethral resection of the fibrous tissue and various laser treatments. [**2**]

During the recent years, various authors presented the bipolar electro-surgical technology as a promising alternative for these patients.

A new development of this technique, the bipolar plasma vaporization (BPV) using the TURis (transurethral resection in saline) system was recently introduced in clinical practice. The equipment used for this approach includes the Olympus UES-40 Surgmaster generator (Olympus, Hamburg, Germany), the OES Pro bipolar resectoscope and the “mushroom” vapo-resection electrode. 

In May 2009, we performed this procedure in the “Sf. Ioan” Clinical Emergency Hospital, Department of Urology as a national premiere in BPH treatment. Subsequently, the technique was also applied in large non-muscle invasive bladder tumors as well as in BNS patients. 

In this retrospective study, we aimed to evaluate our initial experience concerning BPV performed in cases of secondary BNS and to assess the efficiency, safety and short-term postoperative results of this approach.

## Materials and methods

Between May 2009 and May 2010, a total of 30 male patients with a mean age of 71 (range 51 to 89 years old) and secondary BNS underwent BPV and were followed for a period of 6 months. 

BNS was secondary to monopolar TURP in 19 cases, to open surgery for BPH (open prostatectomy) in 8 cases and to radical prostatectomy for prostate cancer in 3 cases. In this last category of patients, there were no signs of local recurrence.

Preoperatively, the investigation protocol included digital rectal examination, blood tests, PSA, urine culture, International Prostate Symptom Score (IPSS), quality of life (QoL) score, maximum flow rate (Qmax), abdominal ultrasonography, post-voiding residual urinary volume (RV) and retrograde uretrography. The inclusion criteria were represented by Qmax < 10 ml/s and IPSS > 19.

**Fig. 1:**
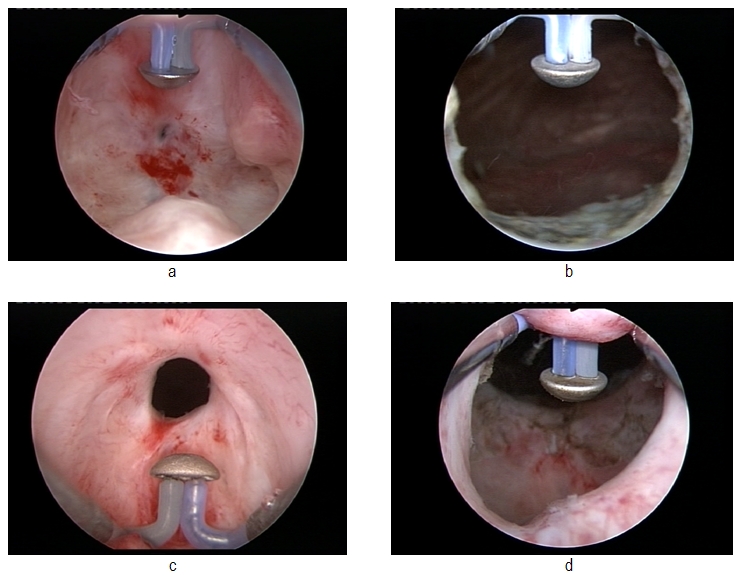
Pre- and postoperative images of the prostatic fossa

The plasma vaporization was successfully performed under spinal anesthesia and using saline continuous flow irrigation. (**Fig. 1**)

**Fig. 2:**
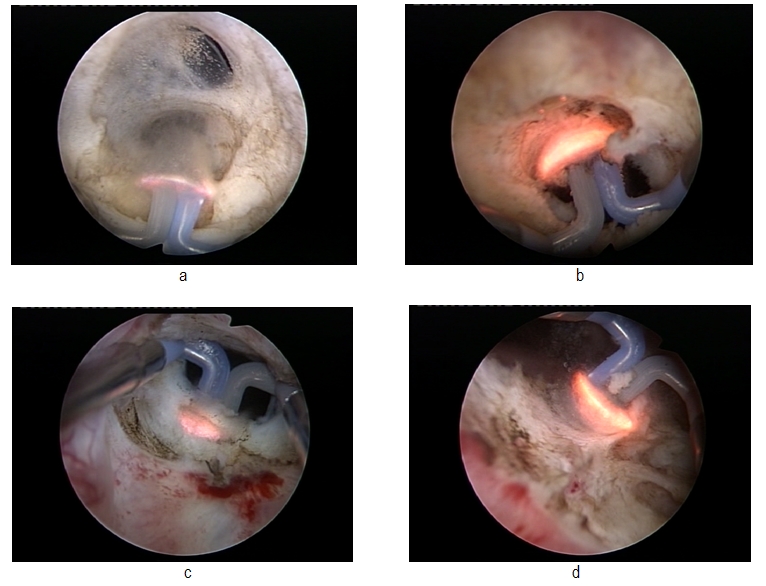
Bipolar plasma vaporization of the fibrous tissue at the bladder neck

The spherical shape new type of electrode displaying a plasma corona on its surface was gradually moved in direct contact with the fibrous tissue (the “hovering” technique), thus producing a virtually blood-less vaporization at 320 W (**Fig. 2**). 

**Fig. 3:**
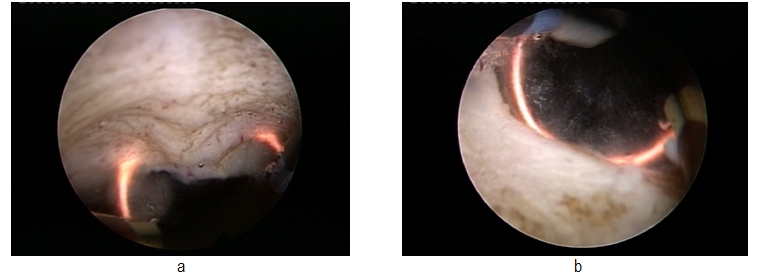
Resection biopsy in bladder neck sclerosis secondary to radical prostatectomy

Several tissue fragments were resected from the bladder neck area for the pathological analysis in all prostate cancer cases. (**Fig. 3**)

All patients were evaluated at 1, 3 and 6 months after surgery by IPSS and QoL scores, Qmax and RV. Control retrograde urethrography and abdominal ultrasonography were performed at 6 months in all cases.

## Results

BPV was successfully performed in all cases. There were no major intra- or postoperative complications. During all procedures, blood loss was insignificant and no patient required blood transfusions. Also, there were no cases of urinary tract infection or sepsis, profound thermal lesions, significant postoperative bleeding or clot retention.

All patients were able to void spontaneously and were continent after catheter removal. On the other hand, four patients presented moderate irritative symptoms (mainly dysuria, urgency and frequency) and were treated conservatively, with no further complications. In all prostate cancer cases, the pathological specimens were negative for malignancy.

The mean operating time was 9 minutes (range 5 to 22 minutes), the mean catheterization period was 18 hours (range 12 to 24 hours) and the mean hospital stay was 24 hours (range 18 to 36 hours).

Preoperatively and at 1, 3 and 6 months after surgery, the mean values for Qmax, were 6.2 ml/s, 23.9 ml/s, 23.8 ml/s and 23.4 ml/s, respectively. At the same moments, the mean RV was 110 ml, 20 ml, 28 ml and 26 ml, respectively. (**Table 1**) There were no aspects suggestive for re-stenosis at the 6 months’ retrograde urethrography.

**Table 1:** Pre- and postoperative parameters

Mean	Preoperative	At 1 month	At 3 months	At 6 months
Qmax	6.2 ml/s (range 3-9.8 ml/s)	23.9 ml/s (range 20.1-27.2 ml/s)	23.8 ml/s (range 19.7-26.8 ml/s)	23.4 ml/s (range 19.5 - 27.1 ml/s)
RV	110 ml (range 45-230ml)	20 ml (range 0- 55 ml)	28 ml (range 0- 65 ml)	26 ml (0-60 ml)
IPSS	22.6 (range 20-27)	3.4 (range 2-6)	3.6 (range 2-7)	3.7 (range 2-7)
QoL	4.1 (range 3-5)	1.2 (range 1-2)	1.3 (range 1-2)	1.3 (range 1-2)

As far as symptom scores were concerned, before surgery, the IPSS and QoL scores were 22.6 and 4.1. During the follow-up, these scores decreased at 1, 3 and 6 months to 3.4 and 1.2, 3.6 and 1.3, and 3.7 and 1.3, respectively. (**Table 1**)

## Discussion

Secondary BNS still remains an important problem in modern urology. In a study by Ying-Huei Lee et al. on 1135 patients which underwent standard TURP, 9.7% of patients developed BNS during a mean follow-up period of 37 months. Small prostates were initially diagnosed in most of these cases [**3**]. 

In another trial by Al-Singary et al. on 900 patients, over a 4-year follow-up period after monopolar TURP, 3.4% of patients developed BNS, with a mean resected prostatic tissue weight of 11 +/- 3.7 g. [**4**] 

The retrograde endoscopic approach represents the main alternative in the treatment of this type of complication. [**5**]

Bipolar plasma vaporization is a relatively new technique, at the beginning as part of the therapeutic armamentarium for lower urinary tract pathology. Despite gaining an increasing acknowledgement as a reliable tool for BPH [**6,7**] and large non-muscle invasive bladder tumors [**8**] treatment, the use of this method in BNS patients was not yet evaluated in clinical trials. 

According to our experience so far and to the results of the present study, there are some important advantages provided by BPV in the treatment of secondary BNS. 

Subjectively, this type of vaporization did not alter the visual characteristics of the tissues, thus enabling the surgeon to differentiate the fibrous tissue and the muscular fibers of the prostatic capsula with increased accuracy. 

Technically, the basis of BPV is represented by the ability of the UES-40 bipolar electrosurgical generator to produce a plasma corona on the surface of the spherical shape “mushroom” type electrode. Plasma vaporization occurs by direct gentle contact with the tissue surface and performs concomitant hemostasis.

The power of the generator can be adapted to tissue characteristics and consistency, thus providing the surgeon with additional technical flexibility: 320 W for fibrous tissue, 280-290 W for the average BPH tissue, 240 W for remaining BPH small fragments close to the capsula or apex and 120-140 W for coagulation. In secondary BNS cases, due to the increased consistency of the scar tissue, a power of 320 W was used during all procedures. This feature provided fast removal of large fibrotic areas.

**Fig. 4:**
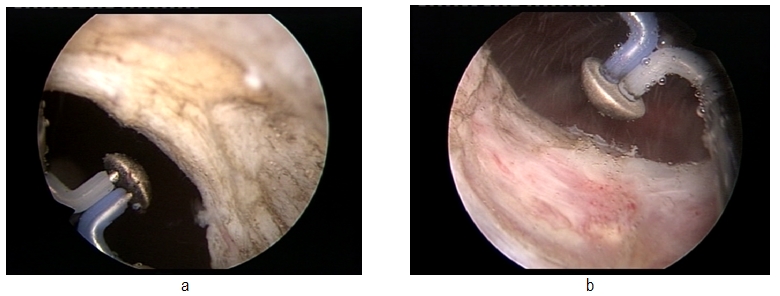
Final aspect of the bladder neck after plasma vaporization

Due to the lack of bleeding, visibility remained excellent throughout the procedure. The vaporization area emphasized a remarkably smooth surface and sharp margins, with no irregularities or debris and no supplementary thermal lesions of the subjacent tissue. (**Fig. 4**)

**Fig. 5:**
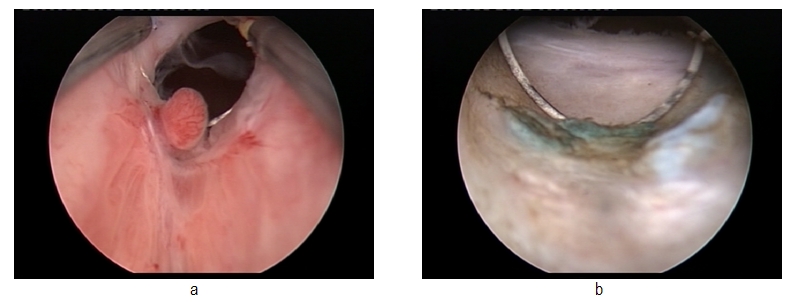
Post- radical prostatectomy bladder neck, before and after bipolar plasma vaporization and resection

The postoperative aspects of the prostatic fossa revealed a large passage, without obstruction, in every case. It is also important to mention that there were no major intra- or postoperative complications, which confirms the safety of the procedure. Another advantage of this technique was represented by the possibility to perform resection biopsy in cases of previous history of prostate cancer by simple changing of the electrode, thus providing a certain pathological analysis for these patients and good functional results. (**Fig. 5**)

As far as the mean operating time was concerned, BPV was superior to PlasmaKineticTM bipolar vaporization [**2**] (9 versus 15 minutes) and similar to laser incision [**9**] (9 versus 7 minutes), thus proving its’ effectiveness in terms of fast scar tissue removal. 

Objectively, our follow-up results seem satisfactory by comparison to the literature data. In a study by Basok et al., secondary BNS patients underwent bipolar vaporization using the PlasmaKineticTM system, a precursor of TURis, after a mean follow-up period of 12.2 months, Qmax increased from 3.4 ml/s before surgery to 16.2 ml/s. [**2**] In our trial, despite the shorter follow-up period, progresses in terms of Qmax were substantial: from the preoperative value of 6.2 ml to 23.4 ml/s at 6 months.

As far as the TURis system is concerned, in a trial by Sevriukov et al. in which only bipolar resection was performed, RV decreased from a mean preoperative value of 92.3 ml to a maximum of 35 ml after surgery, Qmax increased from 8.1 ml/s to 19.8 ml/s and IPSS was reduced from 20.8 to 7.5. [**4**] 

From this perspective, we can say that plasma vaporization constitutes a promising application of the TURis system. Our series emphasized remarkable improvements, as the mean RV decreased from 110 ml to 26 ml at 6 months and progresses in terms of Qmax and IPSS were significant – Qmax increased from 6.2 ml/s to 23.4 ml/s and IPSS decreased from 22.6 to 3.7.

Also, BPV seems comparable to laser incision as well. According to a study by Bach et al. in which 14 patients underwent 2-micron continuous wave laser incision, Qmax increased from 9 ml/s before surgery to 23 ml/s after a 12 months follow-up. The symptom score and QoL score improved from 22 to 8 and from 4 to 1, respectively. [**9**]. It is obvious that our results match these figures, thus proving the efficacy of BPV. On the other hand, the bipolar technology appears to be more advantageous in terms of costs by comparison to laser treatment. [**2**]

Regarding the long-term complications, the re-stenosis rate after endoscopic BNS treatment remains significant according to the literature data, regardless of the applied technique: 13.7% for standard resection [**5**] and 27.5% for monopolar incision of the bladder neck [**4**].

According to published studies, an important advantage of the fibrous tissue instant bipolar vaporization is that it contributes to a decreased recurrent scar tissue formation [**2**]. Also, laser incision was presented to provide disintegration of the fibrous area and secondary reepithelization without scarring. [**10**]

Due to our rather short follow-up period, it may be too early to make a statement in this regard concerning TURis plasma vaporization. However, since there were no urethrographic images suggestive of re-stenosis at 6 months and the progresses in terms of follow-up parameters remained constant, BPV seems to confirm the ability of preventing recurrent fibrosis. This aspect may constitute an interesting endpoint for future studies.

## Conclusions

We may conclude that BPV represents a valuable alternative in the treatment of secondary BNS, with very good efficacy good efficacy, reduced morbidity, fast postoperative recovery and satisfactory follow-up parameters. 

The reduced operating time, catheterization period and hospital stay, as well as the significant postoperative improvements in terms of Qmax, RV, IPSS and QoL score are important advantages of this new technique.

In all these regards, the outcome of BPV compares favorably to monopolar resection or incision, bipolar vaporization or resection as well as to laser incision.

Regarding the re-stenosis rate, BPV seems quite promising but additional extensive studies are required in order to confirm this aspect. Therefore, the long-term results and general viability of the method remain to be established. 
